# Tea as a Beverage

**Published:** 1886-02

**Authors:** 


### TEA AS A BEVERAGE.

Wong Chin Foo, in the Cook.

Use a china or a porcelain pot. If you do use metal let it be tin, new, bright and clean; never use it when the tin is worn out and the iron exposed. If you do you are playing chemist and forming a tannate or tea-ate of iron.

Use black tea. Green tea when good is kept at home. What goes abroad is bad, very bad and horrible Besides containing the two hundred and three adulterations the Chinese philanthropist puts up for the outside barbarian, it is always pervaded by copper dust from the dirty curing pans of the growers.

Infuse your tea. Don't boil it? Place one teaspoonful of tea in the pot and pour over it one and a half cups of boiling water—that is, water really boiling. If your tea is poor, use more. It is cheaper though, to buy good tea at the outset. Put your pot on the back part of the stove, carefully covered, so that it shall not lose its heat and the tea its bouquet. Let it remain there five minutes. Then drink it.

Drink your tea plain. Don't add milk nor sugar. Tea brokers and tea-tasters never do; epicures never do; the Chinese never do· Milk contains fibrin, albumen or some other such stuff and the tea a delicare amount of tannin. Mixing the two makes the liquid turbid. This turbidity, if I remember the cyclopœdia aright is tannate of fibrin or leather. People who put milk in tea are therefore drinking boots and shoes in mild disguise.

